# ANXA6 suppresses the tumorigenesis of cervical cancer through autophagy induction

**DOI:** 10.1002/ctm2.208

**Published:** 2020-10-19

**Authors:** Xin Sun, Yuhan Shu, Mengting Xu, Jiukun Jiang, Liming Wang, Jigang Wang, Dongsheng Huang, Jianbin Zhang

**Affiliations:** ^1^ Department of Oncology People's Hospital of Hangzhou Medical College Hangzhou China; ^2^ College of Biomedical Engineering & Instrument Science Zhejiang University Hangzhou China; ^3^ Department of Emergency Medicine The First Affiliated Hospital School of Medicine Zhejiang University Hangzhou China; ^4^ Department of Physiology National University of Singapore Singapore Singapore; ^5^ Artemisinin Research Center, and Institute of Chinese Materia Medica China Academy of Chinese Medical Sciences Beijing China; ^6^ The First Affiliated Hospital of Southern University of Science and Technology The Second Clinical Medical College of Jinan University Shenzhen People's Hospital Shenzhen China; ^7^ Department of Toxicology School of Public Health Guangxi Medical University Nanning China; ^8^ Key Laboratory of Tumor Molecular Diagnosis and Individualized Medicine of Zhejiang Province People's Hospital of Hangzhou Medical College Clinical Research Institute Hangzhou China

**Keywords:** ANXA6, ATG9A, autophagy, cervical cancer, ERK, mTOR

## Abstract

**Background:**

Autophagy is an intracellular degradation pathway conserved in eukaryotes. ANXA6 (annexin A6) belongs to a family of calcium‐dependent membrane and phospholipid‐binding proteins. Here, we identify ANXA6 as a newly synthesized protein in starvation‐induced autophagy and validate it as a novel autophagy modulator that regulates autophagosome formation.

**Results:**

*ANXA6* knockdown attenuates starvation‐induced autophagy, while restoration of its expression enhances autophagy. GO (gene ontology) analysis of ANXA6 targets showed that ANXA6 interacts with many RAB GTPases and targets endocytosis and phagocytosis pathways, indicating that ANXA6 exerts its function through protein trafficking. ATG9A (autophagy‐related 9A) is the sole multispanning transmembrane protein and its trafficking through recycling endosomes is an essential step for autophagosome formation. Our results showed that ANXA6 enables appropriate ATG9A^+^ vesicle trafficking from endosomes to autophagosomes through RAB proteins or F‐actin. In addition, restoration of ANXA6 expression suppresses mTOR (mammalian target of rapamycin) activity through the inhibition of the PI3K (phosphoinositide 3‐kinase)‐AKT and ERK (extracellular signal‐regulated kinase) signaling pathways, which is a negative regulator of autophagy. Functionally, ANXA6 expression is correlated with LC3 (microtubule‐associated protein 1 light chain 3) expression in cervical cancer, and ANXA6 inhibits tumorigenesis through autophagy induction.

**Conclusions:**

Our results reveal an important mechanism for ANXA6 in tumor suppression and autophagy regulation.

## Graphical abstract

Autophagy is an intracellular degradation pathway conserved in eukaryotes. ANXA6 (annexin A6) belongs to a family of calcium‐dependent membrane and phospholipid binding proteins. Here, we identify ANXA6 as a newly synthesized protein in starvation‐induced autophagy and validate it as a novel autophagy modulator that regulates autophagosome formation. ANXA6 knockdown attenuates starvation‐induced autophagy and restoration of its expression enhances autophagy. GO (gene ontology) analysis of ANXA6 targets showed that ANXA6 interacts with many RAB GTPases and targets endocytic and phagocytosis pathways, indicating that ANXA6 exert its function through protein trafficking. ATG9A (autophagy related 9A) is the sole multi‐spanning transmembrane protein and its trafficking through the recycling endosomes is an essential step for autophagosome formation. Our results showed that under starvation condition, ANXA6 enables appropriate ATG9A trafficking from endosomes to autophagosomes through RAB proteins or F‐actin. In addition, restoration of ANXA6 expression suppresses mTOR (mammalian target of rapamycin) activity through the inhibition of the PI3K (phosphoinositide 3‐kinase)‐AKT and the ERK (extracellular signal‐regulated kinase) signaling pathway, which is a negative regulator of autophagy. Functionally, ANXA6 expression is correlated with LC3 (microtubule‐associated protein 1 light chain 3) expression in cervical cancer and ANXA6 inhibits tumourigenesis through autophagy induction. Our results reveal an important mechanism for ANXA6 in tumor suppression and autophagy regulation.

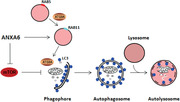

**Abbreviations**
ACTRT1: actin‐related protein T1
AHA: lazidohomoalanine
AKT: protein kinase B
ANXA: annexin
AP: adaptor protein
ATG: autophagy‐related gene
CHX: cycloheximide
CQ: chloroquine
EGFR: epidermal growth factor receptor
ERK: extracellular regulated protein kinase
GAPK: GTPase‐activating protein
IP: immunoprecipitation
LAMP1: lysosome‐associated membrane protein 1
MAPK: mitogen‐activated protein kinase
MS: mass spectrometry
mTOR: mechanistic target of rapamycin
PARP‐1: poly [ADP‐ribose] polymerase 1
PAS: phagophore assembly sites
PI3K: phosphoinositide 3‐kinase
PKC: protein kinase C
tfLC3B: tandem fluorescent‑tagged LC3B
TSC2: tuberous sclerosis 2
ULK1: unc‐51 like autophagy activating kinase 1

## 1 INTRODUCTION

Cervical cancer is the second most common cancer in women and the leading cause of cancer‐related deaths among women in developing countries.^1^, ^2^ Approximately 70% of cervical cancers are caused by HPV (human papilloma virus) 16 and 18 infections. One of the latest opinions about etiopathogenesis^3^ is that HPV leads to the occurrence of cervical cancer through autophagy inhibition. At the early stage of cervical carcinogenesis, HPV infection induces autophagy in cancerous cells^4^, ^5^; conversely, autophagy suppresses HPV infection.^6^ However, at the late stage of cervical carcinogenesis, HPV infection attenuated autophagy^5^; thus, the protective effect of autophagy is lost, and the infectivity of HPV is aggravated to accelerate cervical carcinogenesis. In FIGO stage I‐II cervical squamous cell carcinoma,^7^ the expression levels of autophagy‐related proteins Beclin 1 and LC3 are significantly lower than that in healthy cervical tissue samples, indicating the tumor suppressive effect of autophagy at the early stage of cervical cancer. High levels of autophagy are also a biomarker for good prognosis of cervical cancer.^7^

The autophagic process involves the formation of double‐membrane compartments, called autophagosomes,^8^ which contain cytoplasmic constituents, such as protein aggregates and damaged organelles. Thus, autophagy is regarded as a pathway exclusively regulated by cytosolic processes. However, increasing evidence^9^, ^10^ demonstrates that nuclear transcriptional and epigenetic events also play important roles in autophagy regulation. At the transcriptional level, ATG gene expression can be regulated by some transcription factors and epigenetic changes at histones.^11^ These transcripts can be further regulated at the levels of post‐transcription and translation, such as by noncoding RNAs, RNA‐binding proteins, RNA localization, and decay.^9^ These incidents suggest that *de novo* protein synthesis is implicated in autophagy. In starvation‐induced autophagy, although global protein synthesis is significantly reduced due to the suppression of mTOR (mammalian target of rapamycin), there are still a large number of *de novo* proteins in the autophagic process.^12^ Using bio‐orthogonal metabolic tagging combined with MS (mass spectrometry),^13^ newly synthesized proteins containing AHA (L‐azidohomoalanine) are identified, and functional analysis reveals their involvement in energy metabolism, cell death, cell survival, and so on,^12^ which provides useful insights into the molecular mechanisms and biological functions of autophagy.

Annexins are highly conserved Ca^2+^‐dependent membrane‐binding proteins that exert multiple functions in cellular development and differentiation.^14^, ^15^ According to phylogenetic distribution, they are classified into five groups: A (vertebrates), B (invertebrates), C (unicellular eukaryotes), D (plants), and E (protists). In humans, annexin proteins are conventionally referred to as annexin A1‐11 and A13. Annexins are composed of two principal domains: a variable N‐terminal domain and a conserved C‐terminal core. ANXA6 (annexin A6) has eight homologous annexin repeats, which contain ∼70 highly conserved amino acid residues.^16^ These repeats form a disk with a slight curvature to facilitate the binding of membrane phospholipids in the presence of Ca^2+^.^14^ The Ca^2+^‐binding sites of ANXA6 are located in the annexin repeats 1, 2, 4, 5, 6, and 8.^17^ ANXA6 binds to negatively charged phospholipids in a wide range of intracellular localizations upon activation, especially the plasma membrane and late endosomes/prelysosomes.^15^, ^18^ It participates in membrane trafficking, cytoskeleton organization, cholesterol homoeostasis, and cell adhesion.^18^, ^19^, ^20^ Growing evidence supports^21^ that Ca^2+^ and Ca^2+^‐binding proteins control endocytosis and autophagy because Ca^2+^ originating from lysosomes/late endosomes participates in the converging steps of autophagy and endocytic trafficking. Enrich et al reported^21^ that ANXA6 is identified in the autophagosomes of hepatocytes, indicating a function for ANXA6 in the convergence of endocytic and autophagic vesicles to lysosomes. However, the involvement of ANXA6 in the autophagic and endocytic stages remains poorly understood.

ANXA6 is closely associated with many tumors, as previously described.^18^ However, the functional role of ANXA6 in carcinogenesis is controversial. It has been used as a potential marker for cervical cancer,^22^, ^23^ as several studies have shown the association of ANXA6 with the progression and malignancy of cervical cancer. ANXA6 is reportedly overexpressed in squamous cell cervical carcinoma, especially its N‐terminus.^22^ Its expression in the nucleus of squamous cell cervical carcinoma is stronger than that in squamous cell epithelia. It has been revealed^24^, ^25^ that ANXA6 may act through Ras or Ras/MAPK (mitogen‐activated protein kinase) signaling pathways in cervical cancer, which is mainly mediated by PKC‐α (protein kinase C) or p120GAP (GTPase‐activating protein). P120GAP is the sole GAP known to bind to EGFR (epidermal growth factor receptor) and promote the hydrolysis of Ras‐GTP.^26^ Thus, the interaction of ANXA6 with PKC‐α and p120GAP downregulates the EGFR/Ras signaling pathway. Conversely, interfering with ANXA6 enhances EGF‐induced Ras activity and phosphorylation of ERK (extracellular regulated protein kinase).^27^

In view of the involvement of ANXA6 in autophagy convergence,^21^ ANXA6‐regulated autophagy may be associated with cervical carcinogenesis. Thus, it is important to investigate the role of autophagy in ANXA6‐mediated cervical cancer. We hypothesized that the newly synthesized protein ANXA6 regulates autophagosome formation in starvation‐induced autophagy, which is closely related to cervical carcinogenesis. Here, our study demonstrates that ANXA6 is required for autophagy induction, which promotes appropriate ATG9A^+^ vesicle sorting from endosomes to autophagosomes through RAB proteins or F‐actin. We also showed that ANXA6 correlates with autophagy levels and is downregulated in cervical cancer, while high levels of ANXA6 are associated with improved survival. Functionally, ANXA6‐induced autophagy serves as cell death and enhancing autophagy leads to cervical cancer suppression. These data suggest that ANXA6 expression status may be useful for survival prediction of cervical cancer patients and that ANXA6 could exert its anticancer function through autophagy regulation.

**Headlights**

1. ANXA6 is a newly synthesized protein in starvation‐induced autophagy and a novel autophagy modulator that regulates autophagosome formation.
2. Under starvation, ANXA6 enables appropriate ATG9A trafficking from endosomes to autophagosomes through RAB proteins or F‐actin.
3. ANXA6 suppresses mTOR activity through the inhibition of the PI3K‐AKT and the ERK signaling.
4. ANXA6 inhibits tumorigenesis through autophagy induction.

## 2 MATERIALS AND METHODS

### 2.1 Cell lines and cell culture

HeLa cells stably expressing GFP‐LC3 were kindly provided by Dr. N Mizushima (The University of Tokyo, Japan). The tfLC3 (mRFP‐GFP tandem fluorescence‐tagged LC3 construct) stably transfected L929 cells were from Prof. Shen Han‐Ming's lab (National University of Singapore, Singapore). ANXA6 knockdown HeLa cells were constructed using shRNA specific for ANXA6 lentivirus by us. Other cells were obtained from American Type Culture Collection (ATCC). All cell lines were cultured in DMEM (Sigma, D1152) with 10% fetal bovine serum (HyClone, SV30160.03).

### 2.2 Reagents and antibodies

The chemicals used in our experiments were: rapamycin (Med Chem Express, HY‐10219), CQ (Med Chem Express, HY‐17589), Click‐iT® AHA (Lazidohomoalanine) reagent (Invitrogen, C10289), Tris[(1‐benzyl‐1H‐1,2,3‐triazol ‐4‐yl)methyl]amine (TBTA; Sigma, 678937), (2‐carboxyethyl) phosphine (TCEP; Sigma, C4706), CuSO_4_ (Sigma, 451657), TAMRA alkyne (Invitrogen, T10183), biotin alkyne (Click Chemistry Tools, 1266‐5), U0126 (MCE, HY‐12031), Dulbecco's Modified Eagle's Medium (Invitrogen, 21013), dialyzed fetal bovine serum (Invitrogen, 26400044), 4% formaldehyde (Sigma, F8775) in PBS, 0.5% Triton™ X‐100 (Sigma, T8787) in PBS, and GIBCO^®^ Earle's Balanced Salt Solution (EBSS; Thermo Fisher Scientific 24010043).

The antibodies used in our experiments included: ANXA6 (Abcam, ab31026), ATG9A (Abcam, ab108338), ATG7 (Cell Signaling Technology, 2631), phospho‐ERK (Thr202/Tyr204; Cell Signaling Technology, 4370), ERK (Cell Signaling Technology, 4695), phospho‐mTOR (Ser2448; Cell Signaling Technology, 5536), mTOR (Cell Signaling Technology, 2972), phospho‐PI3K (p85 (Tyr458)/p55 (Tyr199); Cell Signaling Technology, 17366), PI3K (Cell Signaling Technology, 3358), TSC2 (Cell Signaling Technology, 3635), phospho‐AKT (Cell Signaling Technology, 4060), AKT (Cell Signaling Technology, 9272), phospho‐RPS6 (Ser235/236; Cell Signaling Technology, 2211), RPS6 (Cell Signaling Technology, 2217), β‐actin (Sigma, A5441), α‐tubulin (Sigma, T6199), F‐actin (Abcam, ab205), GFP (Cell Signaling Technology, 2555), GFP‐Trap Agarose (ChromoTek, gta‐10), LC3 (Sigma, L7543), P62 (Sigma, P0067), LAMP1 (Cell Signaling Technology, 9091), PARP‐1 (Cell Signaling Technology, 9542), cleaved caspase‐3 (Cell Signaling Technology, 9661), RAB5 (Santa Cruz Biotechnology, sc‐46692), and RAB11 (Proteintech, 15903‐1‐AP).

### 2.3 Chemical metabolic labeling of *de novo* proteins by AHA

HeLa cells were labeled with AHA to detect the newly synthesized proteins as previously described.^12^ AHA (50 µM) was used to lable cells in L‐methionine‐free DMEM (Invitrogen, 21013). To induce autophagy, cells were starved for 2 h. CHX (cycloheximide, 10 µM) was added to inhibit protein synthesis. The AHA‐incorporated proteins were enriched through a click reaction with TAMRA alkyne or alkyne‐bearing biotin. Affinity purification was further performed through adding 50 µL streptavidin beads (Sigma‐Aldrich, S1638). The enriched proteins were either visualized using gel electrophoresis or subjected to iTRAQ labeling for protein identification and quantification using LC‐MS/MS (liquid chromatography‐tandem MS).

### 2.4 Short hairpin RNA and plasmids transient transfection

According to the manufacturer's protocol, the shRNA (short hairpin RNA) targeting *ANXA6* (Santa Cruz Biotechnology, sc‐29688‐V), the shRNA targeting *Atg7* (Santa Cruz Biotechnology, sc‐41447‐V), GFP‐ANXA6 (GeneBio, EX‐A0185‐M98‐B), or GFP‐WIPI2 (WD repeat domain, phosphoinositide interacting 2; GeneBio, EX‐V0950‐M98‐B) were transfected into HeLa cells using Lipofectmine™ 3000 (Invitrogen, L3000015) and then followed by EBSS treatment.

### 2.5 Western blotting

After indicated treatment, cells were harvested by scraping and washed with PBS. Then, cells were lysed in lysis buffer with proteinase inhibitor (P0013, Beyotime). Equal amounts of proteins were prepared for separation by SDS‐polyacrylamide gels and then transferred onto a polyvinylidene fluoride membrane (PVDF, Bio‐Rad, 1620184). After blocking, the membrane was probed with indicated primary and secondary antibodies, developed with the ECL (chemiluminescence) and photographed using Image Quant LAS 4000 system (GE Healthcare).

### 2.6 Immunofluorescence staining and confocal microscopy

HeLa cells stably expressing GFP‐LC3 or L929 cells stably expressing tfLC3 were first seeded to a coverglass slide chamber. After the indicated treatment, cell fluorescence was examined using Leika TCS SP5 Confocal. For immunofluorescence staining, cells were first fixed in 4% paraformaldehyde. After permeabilization by 0.25% Triton X‐100, cells were probed with indicated primary antibodies and fluorochrome‐conjugated secondary antibodies, respectively. Finally, cell fluorescence was determined using confocal microscope.

### 2.7 Immunoprecipitation assay

Briefly, HEK293 cells were lysed on ice for 30 min with the IP (immunoprecipitation) buffer (Beyotime, P0013) with protease inhibitor. Cell lysates were prepared and incubated with RAB5 or RAB11 antibodies overnight with gentle rocking at 4°C, respectively. And then, the immunoprecipitates were washed and boiled in sample buffer. Immunoblotting was performed to analyze the precipitated proteins.

### 2.8 Immunohistochemistry assay

IHC (immunohistochemistry) staining of human cervical cancer samples was performed using anti‐ANXA6 and anti‐LC3 antibodies. A scoring system was used to determine signals in tumor cells from 0 to 3. 0 = no signal, 1 = weak signal, 2 = intermediate signal, and 3 = strong signal. The scores were calculated by multiplying the intensity of signals with the percentage of positive cells.

### 2.9 MS analysis

HEK293 cells were first overexpressed with GFP‐ANXA6 and then treated with EBSS starvation for 2 h. Control cells and starved cells were lysed in IP buffer (Beyotime, P0013) and pulldown was performed using GFP‐Trap Agarose (ChromoTek, gta‐10), respectively. The pulldown samples were separated on 10% (w/v) SDS‐PAGE gels through electrophoresis. After staining with coomassie brilliant blue, the separated protein bands were excised, evaporated using acetonitrile, and resuspended in 1% (v/v) formic acid. After trypsin digestion, samples were injected into the mass spectrometer (Model LTQ, Thermo Fisher Scientific, Waltham, MA, USA). The size of the trap and analytical column was 75 µm x 250 mm, respectively, and both were packed with ChromXP C18‐CL, 3 µm (Eksigent, Germany). Using the high pressure liquid chromatography, these proteolytic peptides were gradient eluted into the mass spectrometer at a flow rate of 0.3 µL/min. By targeting MS/MS scans, the MS/MS data were collected on the 10 most abundant ions occurring in the MS scan. The results are retrieved by Proteome Discoverer according to the following conditions.

**Table:** 

Type of search	MS/MS ion search
Enzyme	Trypsin
Fixed modifications:	Carbamidomethyl (C)
Variable modifications	Oxidation (M) Acetyl (Protein N‐term)
Mass values	Monoisotopic
Protein mass	Unrestricted
Peptide mass tolerance	±15 ppm
Fragment mass tolerance	±20 mmu
Max missed cleavages	2
Instrument type	ESI‐FTICR

After label‐free relative quantitation, GO (gene ontology), KEGG (kyoto encyclopedia of genes and genomes), and IPA (ingenuity pathway analysis) analyses were performed among the identified proteins by LC‐MS/MS to demonstrate the protein‐protein interaction network. The analysis was performed by Keecloud Biotech Co., Ltd. (Shanghai, China).

### 2.10 Colony formation assay

HeLa cells were first seeded in 6‐well plate and then treated with autophagy inducers rapamycin or EBSS starvation. After that, cells were continued to culture until colony formation. After methanol fixation, Gentian Violet was used to stain those surviving colonies, and visible colonies (≥50 cells) were calculated and analyzed.

### 2.11 Detection of cell death

Under phase‐contrast microscopy, cellular morphological changes were photographed. Cell death was quantitatively analyzed using Pacific Blue™ Annexin V staining coupled with flow cytometry (BD Biosciences). Two apoptosis markers PARP‐1 and Caspase‐3 were examined using western blotting.

### 2.12 *In vivo* xenograft tumor model

BALB/c nude mice (female, 4 weeks old) were purchased from the Institute of Zoology, Zhejiang Chinese Medical University. A suspension containing 5 × 10^6^ HeLa or *ANXA6* knockdown HeLa cells was subcutaneously (s.c.) injected into the right flanks of the nude mice to construct two different types of tumor models, respectively. After 1 week, tumor‐bearing mice were randomly divided into two groups: PBS treatment and rapamycin treatment (5 mg/kg/per 2 days). A vernier caliper was used to measure the tumor dimensions twice per week. After 30 days tumor inoculation, mice were sacrificed and tumors were excised and weighed. According to the criteria outlined in the “Guide for the Care and Use of Laboratory Animals,” all animals received humane care. All experiments were conducted following the official recommendations of the Chinese Zoological Society.

### 2.13 Statistical analysis

All western blotting data and image data presented were representative of three independent experiments. The numeric data were presented as mean ± S.D. and analyzed using Student's *t* test. Image J was used for colocalization analysis and the Pearson's correlation coefficient was calculated and analyzed using Student's *t* test. At least 10 cells of one image and three images from each group were chosen for Image J analysis.

## 3 RESULTS

### 3.1 ANXA6 is a newly synthesized protein involved in autophagy induction in cervical cancer

**FIGURE 1:**
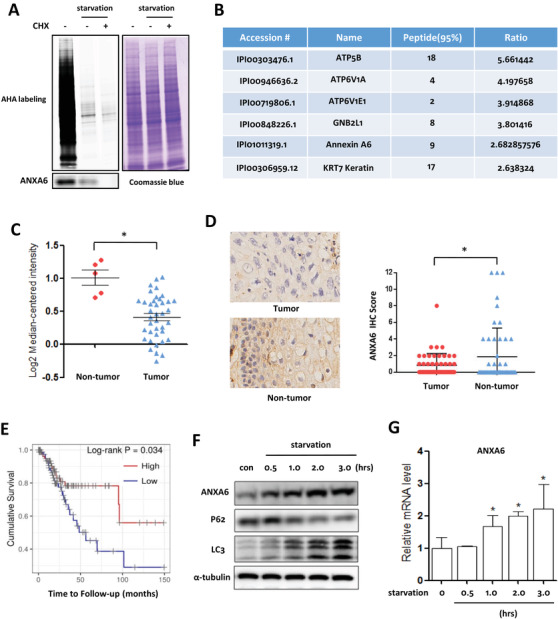
ANXA6 is a newly synthesized protein in starvation‐induced autophagy in human cervical cancer. A, Identification of *de novo* proteins using AHA labeling. HeLa cells were labeled with AHA (50 µM) under starvation in the presence or absence of CHX (10 µM). Intensity of AHA‐labeled proteins was detected in gel fluorescence. The newly synthesized ANXA6 protein was detected using western blotting. B, The representative proteins were shown in database by LC‐MS/MS, including ANXA6. C, D, ANXA6 was downregulated in both mRNA and protein levels in human cervical cancer tissues when compared with that in noncancer tissues. The mRNA levels of *ANXA6* were obtained from Oncomine database (total 45 samples). IHC analysis of ANXA6 expression was performed in 100 human cervical cancer patients from Shanghai Putuo District People's Hospital. * *P <* .05 E, Low expression of ANXA6 was correlated with poor survival in human cervical cancer patients. The data were obtained from TIMER database. F, G, The levels of ANXA6 were increased with time under starvation. HeLa cells were starved with EBSS for autophagy induction

To profile *de novo* protein synthesis during autophagy, the azide‐tagged methionine analogue AHA (L‐azidohomoalanine) is incorporated into protein synthesis.^12^, ^28^ Under 2 h starvation, there was still a lower signal intensity in Figure 1A. After the click reaction, we performed quantitative proteomics and identified 937 newly synthesized proteins. Representative proteins are listed in Figure 1B, and includes ANXA6.^12^ Among them, the functional roles of ATP5B (ATP synthase, H^+^ transporting, mitochondrial F1 complex, and beta polypeptide) and GNB2L1 (guanine nucleotide‐binding protein [G protein], and beta polypeptide 2‐like 1) have been validated in the autophagic process.^12^, ^29^ The newly synthesized ANXA6 protein was also detected using western blotting (Figure 1A).

Next, we analyzed the expression levels of ANXA6 in human cervical cancer. Using the Oncomine database, we first analyzed *ANXA6* mRNA levels in tissue samples from 45 cervical cancer patients and found that it was significantly downregulated in tumor tissues (Figure 1C). Next, we conducted IHC analysis of ANXA6 protein levels in 100 human cervical cancer patients from Shanghai Putuo District People's Hospital. Compared with nontumor tissues, ANXA6 expression was significantly decreased in tumor tissues (Figure 1D). In addition, data from the TIMER (Tumour Immune Estimation Resource) showed that low *ANXA6* mRNA expression in cervical cancer was significantly associated with poor survival (Figure 1E). These results further confirmed the association between ANXA6 and cervical carcinogenesis. In starvation‐induced autophagy, there was an increase in ANXA6 protein levels with time (Figure 1F). Real‐time PCR results showed that starvation led to increased *ANXA6* mRNA levels (Figure 1G), in accordance with its protein synthesis.

### 3.2 Requirement of ANXA6 in the autophagic process

**FIGURE 2:**
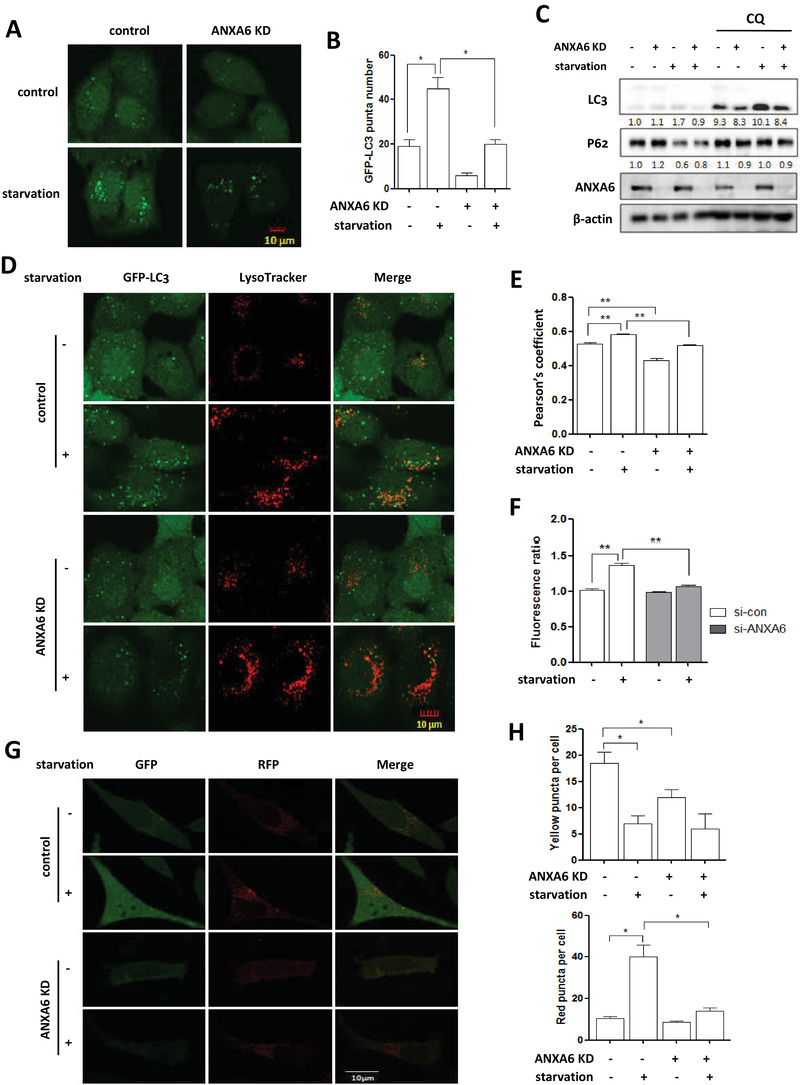
Knockdown of *ANXA6* decreases autophagy levels under starvation. A, B, Hela cells with GFP‐LC3 expressing were starved for 2 h and then cells were examined by confocal microscopy (scale bar 10 µm). The number of GFP‐LC3 puncta was counted and statistically analyzed. * *P* < 0.05 C, HeLa cells were transfected with scrambled or *ANXA6* siRNA for 48 h and then starved in the presence or absence of CQ (20 µM). D, E, HeLa cells with GFP‐LC3 expressing were treated with EBSS for 2 h. After LysoTracker staining (50 nM, 30 min), cells were examined using confocal microscopy (scale bar 10 µm). The Pearson's coefficient was calculated and colocalization was statistically analyzed. ** *P* < 0.01 F, L929‐tfLC3 cells were first transfected with scrambled or *ANXA6* siRNA for 48 h and then starved for 2 h. Cells fluorescence was examined by flow cytometry and the fluorescence ratio of RFP to GFP was calculated and statistically analyzed. ** *P* < 0.01 G, H, as in F, confocal microscope was performed to determine the fluorescence intensity (scale bar 10 µm). The number of yellow puncta (GFP^+^RFP^+^) versus red puncta (GFP^−^RFP^+^) was calculated and statistically analyzed. * *P* < 0.05

In view of Ca^2+^‐binding proteins regulating autophagy,^30^, ^31^ we thus proceeded to test the possible involvement of ANXA6 in autophagy regulation. As shown in Figure 2A and B, *ANXA6* knockdown with siRNA led to a significant decrease in the formation of GFP‐LC3 puncta in HeLa cells treated with EBSS starvation. Moreover, the autophagic flux level decreased in *ANXA6* knockdown cells under starvation, accompanied by a lower LC3 level and increased P62 (one autophagy substrate) (Figure 2C). Additionally, in rapamycin‐induced autophagy, the decrease in autophagy level due to *ANXA6* knockdown is also shown in Figure S1. These results clearly suggest that ANXA6 plays a critical role in autophagy induction.

ANXA6 is reported to exert a function in vesicle fusion through binding to negatively charged phospholipids of membranes.^32^, ^33^ Here, we also determined the effect of ANXA6 on the fusion of autophagosomes and lysosomes. In EBSS‐treated cells, we observed increased localization of GFP‐LC3 in lysosomes (Figure 2D and E), indicating the enhancement of autophagosome‐lysosome fusion. However, in *ANXA6* knockdown cells, their colocalization appeared to be decreased (Figure 2D and E). In addition, tfLC3B (mRFP‐GFP tandem fluorescent‐tagged LC3B) was also used to examine autophagosome and lysosome fusion.^34^ In the acidic lysosome environment, GFP‐LC3 is degraded, while RFP‐LC3 is stable. As shown in Figure 2F and H, EBSS starvation increased RFP puncta instead of GFP puncta, while *ANXA6* knockdown attenuated this increase. Similar results were also observed in rapamycin‐treated cells, in which *ANXA6* knockdown impaired the enhanced formation of autolysosomes (Figure S2). Therefore, ANXA6 seems to be a new Ca^2+^ effector that regulates the converging steps of autophagy.

### 3.3 Identification of ANXA6 targets by quantitative proteomics

**FIGURE 3:**
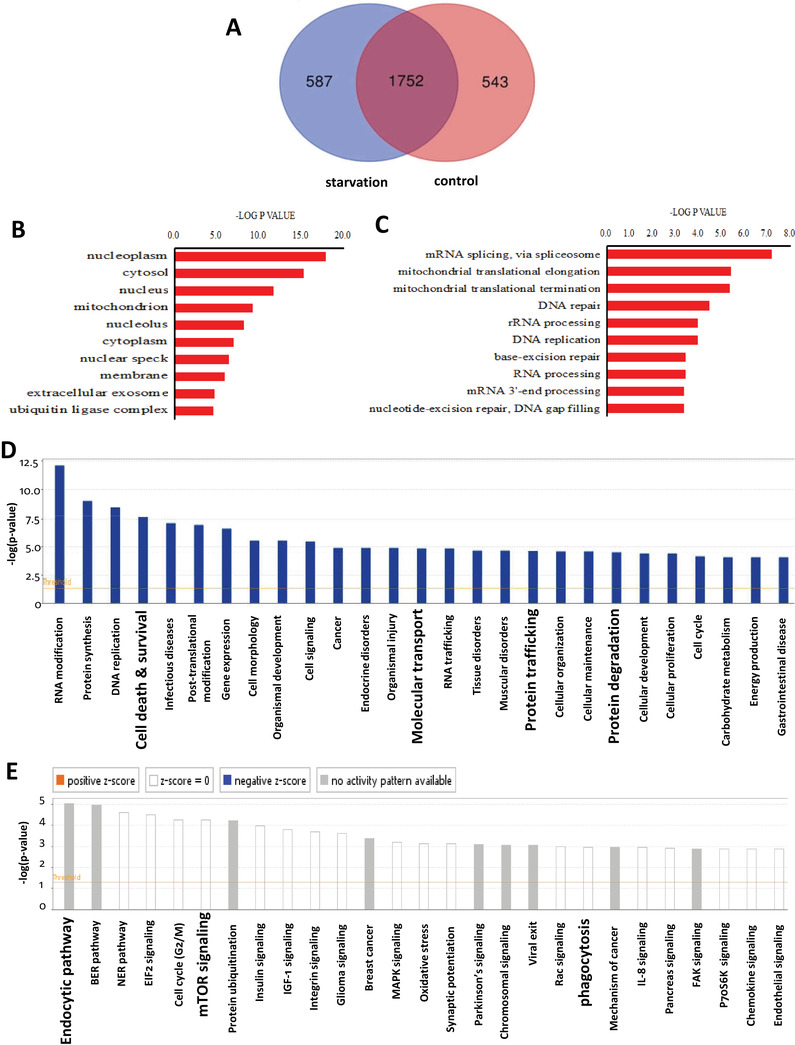
Quantitative proteomics reveals ANXA6‐specific target proteins and involved pathways. A, Total 587 proteins were profiled as target proteins of ANXA6 under starvation. B, GO analysis of CC (cellular component) localization of the ANXA6 targets. C, The top regulated BPs (biological processes) of ANXA6 targets were shown according to their ranking. D, Top molecular and cellular functional classes to which the ANXA6‐targeted proteins are associated. E, Top canonical pathways that the ANXA6 protein targets are significantly over‐represented

To reveal the molecular mechanism of ANXA6‐induced autophagy, quantitative proteomics was performed to identify the targets of ANXA6. HEK293 cells were first transfected with GFP‐ANXA6 and then starved in EBSS for 2 h before lysis. The lysate was then subjected to affinity enrichment using GFP‐fusion beads. The beads were first thoroughly washed and then digested using trypsin. LC‐MS/MS was used to pool and analyze the derived peptides and finally the target proteins were identified and quantified. To differentiate specific targets, a highly stringent cutoff threshold was applied to minimize potential false‐positive targets. Each identified protein was subjected to statistical tests and only proteins identified with *P*‐values less than 0.05 were considered statistically reliable hits. In our study, a total of 2295 proteins from the control group and 2339 proteins from the starvation‐treated group were successfully identified and quantified (Figure 3A). Among them, we identified 587 proteins as the specific targets of ANXA6 under starvation (Table S1).

Subsequently, we performed GO analysis of the ANXA6 targets under starvation. It was shown that these targets were broadly distributed in different parts of the cell, especially in the cytosol, nucleus, mitochondria, and membrane (Table S1). The distribution of the enrichment ratios of these proteins is presented as a histogram in Figure 3B. GO analysis also showed that ANXA6 targets are involved in many biological processes (Figure 3C), such as mRNA splicing, mitochondrial elongation, DNA replication, and repair. Accordingly, they exert various biological functions, including RNA and DNA modification, protein trafficking, molecular transport, protein synthesis and degradation, cell death, and survival (Figure 3D and Figure S3A). In Figure 3E, Figure S3B, and Table S2, GO and KEGG analysis of pathways demonstrated that ANXA6 may exert its effects through the endocytic pathway, mTOR signaling, eukaryotic initiation factor 2 signaling, MAPK signaling, oxidative stress, and phagocytosis of proteins.

### 3.4 ANXA6 regulates ATG9A^+^ vesicle trafficking through RAB proteins or F‐actin

**FIGURE 4:**
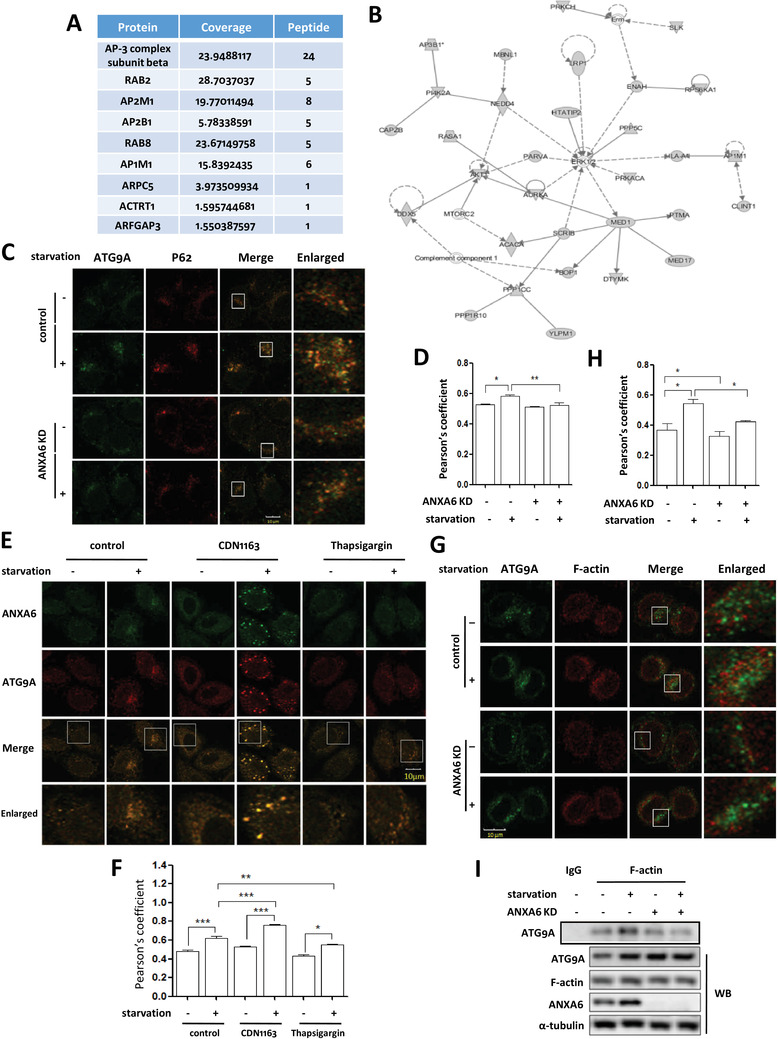
ANXA6 targets molecular transporters to regulate the formation of autophagosome. A, The representative target proteins of ANXA6 identified by LC‐MS/MS in cells (sorted by average enrichment ratios). B, IPA reveals that ANXA6 affects protein trafficking. All proteins shown were identified as specific targets of ANXA6. C, D, The colocalization of ATG9A with P62 in HeLa cells was examined under starvation (scale bar 10 µm). The Pearson's coefficient was calculated and statistically analyzed. * *P* < 0.05 ** *P* < 0.01 E, F, Hela cells were starved in EBSS for 2 h in the presence or absence of CDN1163 (an activator of Ca^2+^‐ATPase) or thapsigargin (an inhibitor of Ca^2+^‐ATPase). The colocalization of endogenous ATG9A with ANXA6 was determined by confocal microscopy (scale bar 10 µm). The Pearson's coefficient was calculated and statistically analyzed. * *P* < 0.05 ** *P* < 0.01 *** *P* < 0.001 G, H, The colocalization of ATG9A with F‐actin was examined under starvation (scale bar 10 µm). The Pearson's coefficient was calculated and statistically analyzed. * *P* < 0.05 I, IP assay was also conducted to determine the interaction between ATG9A and F‐actin

GO analysis showed that ANXA6 targets endocytic and phagocytic pathways. Under normal conditions, 15 RAB proteins of ANXA6 targets in the database are listed in Figure S3C, including RAB2, RAB5, RAB7, and RAB11. RABs belong to small GTPases and have critical role in molecular trafficking.^35^, ^36^ Under starvation, eight representative proteins of ANXA6 targets were selected and listed (Figure 4A), such as Rab GTPases RAB2 and RAB8, AP complexes (adaptor protein), and ACTRT1 (actin‐related protein T1). AP complexes, also known as vesicle coat components, exert an important function in protein transport in membrane traffic pathways.^37^ As shown in Figure 4B, IPA also suggested that ANXA6 exerts its function through protein trafficking. It has been known that several RAB GTPases are involved in autophagy regulation.^38^, ^39^ An RAB‐conversion mechanism is revealed to involve in endocytosis or phagocytosis in which signals are seamlessly transduced to form endosomes or phagosomes. For example, RAB2 transports ATG9^+^ vesicles derived from Golgi apparatus to the PAS (phagophore assembly site) .^40^ RAB2 interacts with ATG9 and facilitates the colocalization of ATG9 and ULK1 (unc‐51 like autophagy activating kinase 1), resulting in ULK1 acquisition and activation to facilitate phagophore formation.^40^

Previous studies have demonstrated that ATG9^+^ vesicle derived from plasma membrane or recycling endosome contributes to autophagosome biogenesis.^41^, ^42^ As shown in Figure 4C and D, EBSS starvation enhanced the colocalization of ATG9A and autophagy substrate P62, but *ANXA6* knockdown attenuated their interaction, indicating the role of ANXA6 in the trafficking of ATG9A to the site of autophagosome biogenesis. This was further confirmed by the enhanced colocalization of ANXA6 and ATG9A under starvation (Figure 4E and F). In addition, their colocalization was found to depend on Ca^2+^ (Figure 4E and F) because ANXA6 is a highly conserved Ca^2+^‐dependent membrane‐binding protein.^14^, ^15^ In the presence of CDN1163, an allosteric activator of sarco/endoplasmic reticulum Ca^2+^‐ATPase, the colocalization of ANXA6 and ATG9A was enhanced under starvation. However, in the presence of thepsigarin, a highly potent inhibitor of the sarco‐endoplasmic reticulum Ca^2+^‐ATPase, their enhanced interaction was attenuated under starvation. ANXA6 interacts with cytoskeleton components through an F‐actin binding domain to remodel the plasma membrane.^43^ The actin cytoskeleton has a critical role in ATG9A^+^ vesicle motion.^44^ In our study, under starvation, the localization of ANXA6 or ATG9A and F‐actin was increased, while knockdown of *ANXA6* attenuated their interaction (Figure 4G and H; Figure S4). In addition, IP results also showed the enhanced interaction of ATG9A and F‐actin poststarvation and the requirement of ANXA6 for their interaction (Figure 4I). These data suggest that ANXA6 may control autophagosome formation through actin and potentially via ATG9A sorting at the endosomal level.

### 3.5 ANXA6 regulates ATG9A sorting from recycling endosomes to form autophagosomes

**FIGURE 5:**
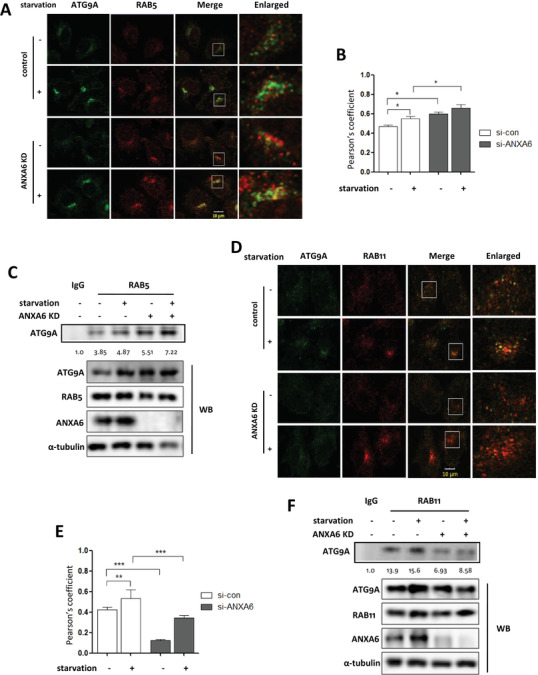
Knockdown of *ANXA6* attenuates ATG9A trafficking through the recycling endosomes. A, B, Confocal pictures showed the colocalization of ATG9A with RAB5 in starved HeLa cells with *ANXA6* knockdown (scale bar 10 µm). The Pearson's coefficient was calculated and statistically analyzed. * *P* < 0.05 C, IP assay was performed to determine the interaction between ATG9A and ANXA6. HEK293 cells with *ANXA6* knockdown were transfected with pcDNA3.1‐ATG9A and then starved with EBSS. Cell lysates were prepared for IP assay. D, E, The colocalization of ATG9A with RAB11 in ANXA6 knockdown cells was determined under starvation. (scale bar 10 µm). The Pearson's coefficient was calculated and statistically analyzed. ** *P* < 0.01 *** *P* < 0.001 F as in C, IP assay was conducted to examine the interaction between ATG9A and RAB11 in ANXA6 knockdown cells

ATG9A, a transmembrane protein, functions in membrane trafficking to the preautophagosome structures or autophagosomes.^45^, ^46^ In mammalian cells, ATG9A traffics among Golgi, various endocytic vesicles, and autophagosomes.^47^, ^48^ In the N‐terminal cytosolic stretch, ATG9A has sorting motifs for trafficking.^49^, ^50^ Through interaction with the AP complexes, it is sorted and packed into a transport vesicle as cargo.^51^ These ATG9‐containing vesicular carriers are transported toward sites of autophagosome formation. It was revealed that ANXA6 interacts directly with TPC1/2 (two pore channels) of the late endosomes that induce the endocytic or autophagic trafficking pathways,^52^ or with the proton pump H^+^ ATPaseV0A2.^53^ We first knocked down *ANXA6* in HeLa cells and then starved cells to understand the regulation of autophagosome formation by ANXA6. We next analyzed the localization of ATG9A with an endosomal marker and observed that *ANXA6* knockdown led to increased ATG9A accumulation in the early endosomes (RAB5^+^ vesicles) under starvation (Figure 5A and B). As shown in Figure 5C, the IP assay also showed that knockdown of *ANXA6* enhanced the interaction of ATG9A with RAB5. This suggested that the ANXA6‐dependent actin mechanism transports ATG9A through early endosomes. As a result, the disturbed ATG9A trafficking affected autophagosome formation.

Recent studies^44^, ^54^ showed that ATG9A has an important function in autophagosome biogenesis with its transient localization to recycling endosomes. We also observed that starvation led to ATG9A vesicle accumulation in the recycling endosomes using RAB11 as a marker (Figure 5D and E). RAB11 exerts its function in regulating ATG9A trafficking to autophagic compartments from the plasma membrane.^42^, ^55^ However, *ANXA6* knockdown decreased ATG9A localization in RAB11‐positive vesicles. Consistently, the IP assay also showed that *ANXA6* knockdown weakened the interaction between ATG9A and RAB11 (Figure 5F). These data suggest that ANXA6 controls ATG9A trafficking to recycling endosomes.

### 3.6 Restoration of ANXA6 expression induces autophagy

**FIGURE 6:**
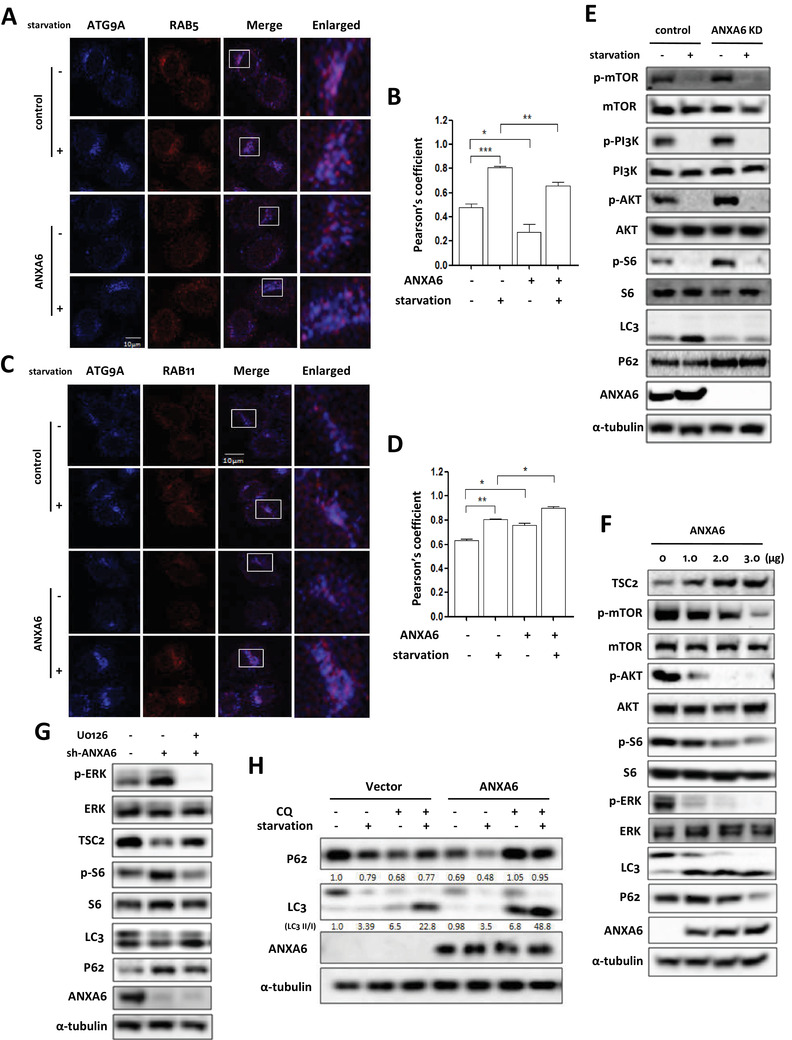
ATG9A trafficking by ANXA6 is required for the formation of autophagosome. A, C, GFP‐ANXA6 was transiently transfected into HeLa cells with *ANXA6* knockdown and then cells were starved in EBSS for 2 h. Confocal microscope was used to determine the colocalization of ATG9A with RAB5 or RAB11 (scale bar 10 µm). The Pearson's coefficient was calculated and statistically analysed in B, D. * *P* < 0.05 ** *P* < 0.01 *** *P* < 0.001 E, HeLa cells with or without *ANXA6* knockdown were starved for 2 h. Cells were harvested and lysed for western blotting. α‐tubulin served as loading control. F, HeLa cells with *ANXA6* knockdown were transiently transfected with different amount of ANXA6. Cell lysates were prepared for western blotting to determine the expression levels of the indicated proteins. α‐tubulin was used as loading control. G, HeLa cells with *ANXA6* knockdown were treated with ERK inhibitor U0126 (10 µM) for 2 h. Cells were harvested for western blotting to determine the indicated proteins expression. H, HeLa cells with *ANXA6* knockdown were first transiently transfected with GFP‐ANXA6 and then starved in EBSS for 2 h in the presence or absence of CQ (20 µM). Cells were harvested and lysed for western blotting and α‐tubulin served as loading control

We restored the expression of ANXA6 in knockdown cells to assess the intracellular localization of ATG9A to confirm the role of ANXA6 in ATG9A trafficking. As shown in Figure 5A and B, increased accumulation of ATG9A was observed at the early endosomes (RAB5^+^ vesicles) under starvation during *ANXA6* knockdown. The effect of ANXA6 downregulation on ATG9A sorting could be rescued by transiently expressing ANXA6, as seen by reduced colocalization of ATG9A‐RAB5 (Figure 6A and B), and increased colocalization of ATG9A‐RAB11 under nutrient‐starved conditions (Figure 6C and D). These results indicate that restoration of ANXA6 expression leads to the trafficking of ATG9A from the early endosomes to the recycling endosomes for autophagosome formation.

GO analysis revealed that ANXA6 targets were associated with mTOR signaling (Figure 3E). mTOR, an evolutionarily conserved protein kinase, serves as a central regulator of cell growth and is a key negative regulator of autophagy.^56^ Nutrient starvation activates autophagy through suppression of mTOR signaling and activation of the ULK1 complex in eukaryotic cells.^57^, ^58^ To reveal the role of mTOR signaling in autophagy regulation by ANXA6, we conducted RNA interference to knock down *ANXA6* expression and found that the PI3K (phosphoinositide 3‐kinase)‐AKT (protein kinase B)‐mTOR signaling pathway was activated by *ANXA6* knockdown (Figure 6E), accompanied by an increase in the phosphorylation levels of PI3K, AKT, mTOR, and S6. Consequently, cells with *ANXA6* knockdown exhibited lower LC3 levels and higher P62 levels (Figure 6E and Figure S5), indicating decreased autophagy levels due to mTOR activation. Conversely, when we restored the expression of ANXA6 in knockdown cells, the phosphorylation levels of AKT, mTOR, and S6 decreased with increasing ANXA6 levels (Figure 6F).

In addition, IPA analysis showed that ERK signaling was involved in ANXA6‐induced autophagy (Figure 4B). Based on the literature, the ERK pathway can cross‐activate PI3K‐mTOR signaling by regulating TSC2 (tuberous sclerosis 2) and mTOR.^59^ Through the posttranslational repression of TSC2, ERK exerts an important function in TSC progression. ERK‐mediated phosphorylation results in the dissociation of TSC1 and TSC2 and further weakens the ability of TSC2 to inhibit mTOR signaling.^59^, ^60^ Thus, we determined the levels of ERK in *ANXA6* knockdown cells. As shown in Figure 6G, *ANXA6* knockdown increased the phosphorylation levels of ERK and decreased TSC2 protein levels. ANXA6 overexpression attenuated the increase in phosphorylation levels of ERK. Moreover, ERK inhibitor increased TSC2 protein levels and attenuated mTOR activity in *ANXA6* knockdown cells (Figure 6G). The above results demonstrate that ANXA6 may inhibit mTOR function by blocking ERK and PI3K‐AKT signaling.

As a result, mTOR inhibition led to increased LC3 levels and decreased P62 levels (Figure 6F and G; Figure S5). Using chloroquine (CQ, an autophagy inhibitor), we also measured autophagy flux‐level changes during restoration of ANXA6 expression. As shown in Figure 6H, under EBSS starvation, LC3 levels greatly increased in the presence of CQ. When restoring *ANXA6* in knockdown cells, high levels of LC3 were further increased (Figure 6H), indicating enhanced autophagy flux. Meanwhile, ANXA6 restoration reduced P62 levels and increased autophagy flux. The above results demonstrated that ANXA6 induces autophagy through mTOR suppression.

### 3.7 Decreased autophagy levels by *ANXA6* knockdown lead to aberrant cell growth

**FIGURE 7:**
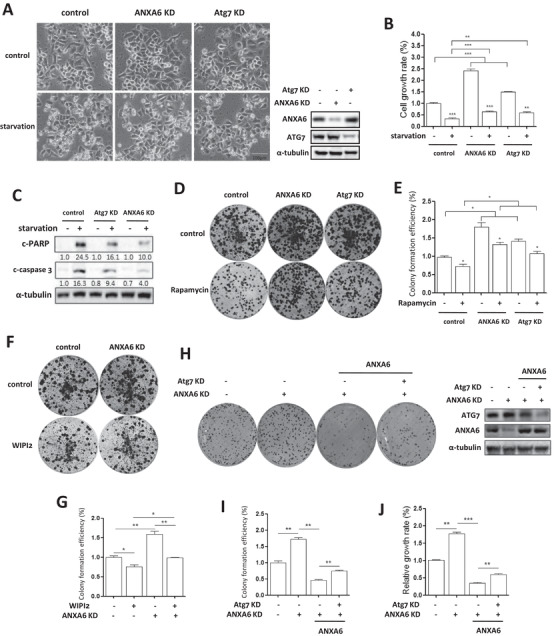
Low levels of autophagy promotes the proliferation of cancer cells. HeLa cells were first transfected with the *ANXA6*‐ or *Atg7*‐specific lentiviral shRNA. After 72 h, cells were cultured in EBSS for 24 h starvation. A, HeLa cells with indicated treatment were examined under an inverted microscope and morphological change of cells was photographed (scale bar 100 µm). Western blotting was used to determine ANXA6 and ATG7 expression. B, Cell proliferation was measured by CCK‐8 (cell counting kit‐8) under starvation and statistically analyzed. ** *P* < 0.01 *** *P* < 0.001 C, Cell lysates were prepared for western blotting to determine the apoptotic markers. D, E, as in A, cells were treated with autophagy inducer rapamycin. Cell proliferation was determined by colony formation assay. A quantitative analysis of the colony numbers was shown in the bar diagram. * *P* < 0.05 F, G, HeLa cells with or without *ANXA6* knockdown were transfected with GFP‐WIPI2 and colony formation assay was performed to measure cell proliferation. * *P* < 0.05 ** *P* < 0.01 H, I, HeLa cells with *ANXA6* knockdown were first restored the expression of ANXA6 and then transfected with the *Atg7*‐specific lentiviral shRNA. Western blotting was conducted to examine the expression levels of ANXA6 and ATG7. Cell proliferation was determined by colony formation assay. ** *P* < 0.01 J, Cell proliferation was measured using CCK‐8 and statistically analyzed. ** *P* < 0.01 *** *P* < 0.001

Next, we sought to determine the biological function of autophagy in cancer cell growth. IHC analysis showed that ANXA6 was lowly expressed in human cervical cancer tissues (Figure 1C), indicating its tumor suppressive effect.^18^ As expected, the morphological changes and CCK‐8 results showed that knockdown of *ANXA6* or *Atg7* in HeLa cells promoted proliferation (Figure 7A and B; Figure S6C), which may be due to lower autophagy levels in knockdown cells. To test this, EBSS starvation was used to induce autophagy^56^ and Annexin V staining showed that cell apoptosis significantly increased under starvation (Figure S6A). However, in starved‐*ANXA6* knockdown cells, quantification of apoptotic events was far less than that in wild‐type cells (Figure S6A). As shown in Figure 7C, western blotting results also showed that starvation reduced cellular apoptosis in *ANXA6* or *Atg7* knockdown cells, accompanied by less cleavage of caspase 3 and PARP‐1 (poly [ADP‐ribose] polymerase 1), two known apoptotic markers. In addition, we tried to restore ANXA6 expression in knockdown cells and found that ectopic expression of ANXA6 led to increased apoptosis when compared with *ANXA6* knockdown cells under starvation using Annexin V staining (Figure S7). Such observations indicate that ANXA6‐induced autophagy serves as a cell death mechanism.

Rapamycin (an mTOR inhibitor) was used to induce autophagy.^61^ As shown in Figure 7D and E and Figure S6B, *ANXA6* or *Atg7* knockdown stimulated cancer cell proliferation to a greater extent than wild‐type cells based on the results of cell growth curves and colony formation assays. Rapamycin treatment induced autophagy and significantly inhibited cancer cell viability and colony formation. Consistently, knockdown of *ANXA6* attenuated the inhibitory effects of rapamycin on cell growth due to reduced autophagy induction. In addition, we also performed genetic induction of autophagy via ectopic expression of WIPI2.^62^ As shown in Figure 7F and G, WIPI2 overexpression significantly inhibited cancer cell colony formation, but *ANXA6* knockdown weakened the suppressive effect of autophagy induction. Moreover, we restored ANXA6 expression in knockdown cells and found that ANXA6 significantly suppressed cell growth and colony formation (Figure 7H‐J and Figure S7). However, when *Atg7* was knocked down, the inhibitory effect of ANXA6 restoration was attenuated, further confirming that autophagy serves as cell death.

### 3.8 *In vivo* tumor suppression by autophagy activation

Finally, we evaluated whether autophagy induction could exert an antitumor effect in xenograft tumor modeling. Wild type or *ANXA6* knockdown HeLa cells were inoculated S.C. into the flank of nude mice. Two different types of xenograft tumor models were established, in which tumor‐bearing mice were divided into two groups: (1) mice treated with PBS and (2) mice treated with rapamycin. The xenograft tumors developed for 4 weeks after injection, and the treatment commenced 1 week postinoculation when the subcutaneous tumor mass formed.

**FIGURE 8:**
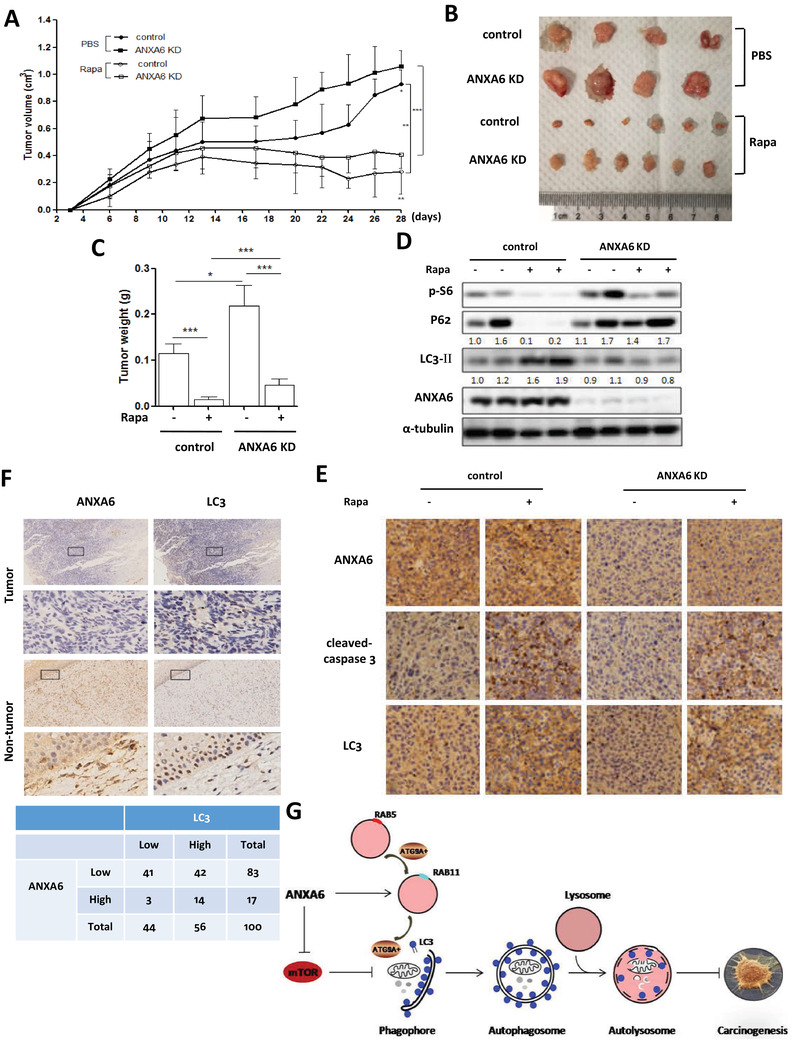
Activation of autophagy suppresses the tumorigenesis of cervical cancer. A, Tumor volumes from each group were measured using a vernier caliper twice per week and then statistically analyzed. * *P* < 0.05, **P < 0.01, *** *P* < 0.001 B, Typical images of the xenograft tumors from different treatment groups were shown. C, The average tumor weight from each group was calculated and statistically analyzed. * *P* < 0.05, *** *P* < 0.001 D, Autophagy markers were examined in tumor tissue from various groups using western blotting. E, Representative images of ANXA6, cleaved‐caspase 3, and LC3 by IHC staining were shown on serial sections of tumors from various groups. F, ANXA6 expression was associated with LC3 level in human cervical tumor and nontumor tissues by IHC staining (upper panel). IHC staining results were summarized in two different cohorts of human cervical cancer specimens (lower panel). G, A schematic model of autophagy induction by ANXA6 in human cervical cancer

Consistently, *ANXA6* knockdown significantly promoted tumorigenesis when compared with wild‐type cells (Figure 8A). These observations were also strengthened by increased tumor weight and larger tumor volume in the *ANXA6* knockdown tumor model after mice were sacrificed (Figure 8B and C). Significant tumor suppression was observed in the rapamycin‐treated groups compared with the PBS‐treated groups, but the tumor suppressive effect was attenuated by *ANXA6* knockdown. As shown in Figure 8D, western blotting results showed that rapamycin treatment induced autophagy in wild‐type tumors instead of *ANXA6* knockdown tumors, accompanied by increased LC3 and decreased P62 levels. This may be attributed to the higher levels of phospho‐S6 in rapamycin‐treated *ANXA6* knockdown tumors (Figure 8D), indicating the activation of mTOR signaling. IHC was used to evaluate the levels of apoptosis and autophagy in the xenograft tumor, and we observed that rapamycin treatment greatly increased caspase 3 cleavage and LC3 level in wild‐type tumors than in *ANXA6* knockdown tumors (Figure 8E). To evaluate the clinical importance of ANXA6‐induced autophagy in cervical cancer, we analyzed the correlation between ANXA6 and LC3 expression levels in human cervical cancer specimens. The levels of ANXA6 and LC3 were determined using IHC staining. In Figure 8F, ANXA6 expression had a significant correlation with LC3 level in these cervical cancer samples. Taken together, these findings markedly indicate that autophagy exerts an anticancer effect.

A schematic model of autophagy induction by ANXA6 in human cervical cancer is shown in Figure 8G. On the one hand, through RAB proteins or F‐actin, ANXA6 enables appropriate ATG9A^+^ vesicle trafficking to autophagosomes from endosomes. On the other hand, ANXA6 expression leads to mTOR suppression and autophagy initiation. ANXA6 exerts an anticancer effect in cervical carcinogenesis through autophagy induction.

## 4 DISCUSSION

In response to starvation, autophagy levels increase to ensure sufficient energy provision for cell survival through degradation of cytoplasmic constituents and organelles. In this process, many proteins are synthesized, which may be involved in autophagy or biological functions of autophagy. Here, we identified a newly synthesized protein, ANXA6, in starvation‐induced autophagy in cervical cancer cells. ANXA6 induction was due to its transcriptional upregulation with increased mRNA levels (Figure 1G), but its upstream modulator was not explored in our study. Autophagy induction was dependent on ANXA6 level, as it was abrogated by *ANXA6* knockdown, while restoration of ANXA6 expression in cells was able to enhance autophagosome formation, confirming that the autophagy process requires the synthesis of ANXA6. In cervical cancer, ANXA6 was downregulated and low levels of ANXA6 were associated with poor survival of cervical cancer patients (Figure 1), indicating that it serves as a tumor suppressor. This was also confirmed by *in vitro* and *in vivo* experiments. The tumor suppressive effect of ANXA6 could be attributed to autophagy induction. Knockdown of *ANXA6* decreased autophagy levels and increased cervical tumor growth, while induction of autophagy significantly suppressed cervical tumorigenesis (Figure 8). These results demonstrate that ANXA6‐induced autophagy exerts an anticancer effect in cervical carcinogenesis.

To reveal the molecular mechanism of ANXA6‐induced autophagy, quantitative proteomics was conducted to profile the targets of ANXA6. Among them, multiple molecules regulating protein trafficking were identified and IPA showed that ANXA6 regulates autophagy mainly through molecule transport and protein trafficking (Figure 3). These molecules include RAB GTPases RAB2 and RAB8, adaptor protein AP complexes, and ACTRT1, enabling the maintenance of starvation‐induced autophagy. Either in endocytosis or phagocytosis, an RAB‐conversion mechanism is involved in the maturation of endosomes or phagosomes.^63^, ^64^ Recently,^65^ growing evidence has demonstrated that members of RAB small GTPases regulate the autophagic process. RAB8 interacts with the autophagy receptor Optineurin and is recruited to PAS to initiate autophagy.^66^ The AP2 complex, together with the RabGAP protein TBC1D5,^67^ regulates the rerouting of ATG9‐containing vesicle toward sites of autophagosome formation. Thus, the identification of multiple target molecules and cooperating responses demonstrates that ANXA6‐dependent autophagosome formation involves many trafficking pathways of converging membrane.

ATG9A is mainly localized to the trans‐Golgi network and the endosomal system,^46^, ^68^ and reportedly moves between them.^48^, ^69^ ATG9A also localizes with ULK1 and ATG16L at recycling endosomes.^42^, ^67^ When autophagy is induced, ATG9A transiently translocalizes to autophagosomes from recycling endosomes through the interaction of its sorting motifs with AP complexes.^67^ In fact, the trafficking of ATG9A among endosomes is also associated with ANXA6, and knockdown of *ANXA6* decreased ATG9A trafficking from early endosomes to recycling endosomes under starvation (Figure 5). As a result, the localization of ATG9A in autophagosomes was also attenuated and autophagosome formation was impaired. As an autophagy modulator, the function of ANXA6 is similar to that of another annexin family member ANXA2.^44^ Unfortunately, ATG9A was not predicted in the MS analysis of ANXA6‐interacting proteins. In our MS data, ANXA6 was found to interact with RAB small GTPases, including RAB2, RAB8, and AP complexes (Figure 4A). These molecular targets are involved in vesicle trafficking to PAS.^40^, ^66^, ^67^ Thus, we speculate that ANXA6 may regulate ATG9A^+^ vesicles to form phagophores through these molecular transporters instead of directly regulating the trafficking of ATG9A‐containing vesicles.

In fact, ATG9 is found to not only be involved in the formation of ILVs (intraluminal vesicles), but is also involved in the localized acidification within amphisomes/autolysosomes.^70^ This means that ANXA6 may exert an effect on the regulation of MVB (multivesicular body) pathways. MVBs contain ILVs and these contents are ubiquitinated and degraded in the lysosome or recycled to cell surface in the endocytic pathway.^71^ This process is achieved through the action of protein complexes named endosomal sorting complex required for transport‐0,I,II, and III.^72^ We also examined the degradation of EGFR, which is internalized via clathrin‐mediated endocytosis.^73^ A significant portion of EGFR is ubiquitinated and sorted to ILVs within the MVB.^74^ Our results showed that ANXA6 was required for the degradation of EGFR (data not shown), suggesting that ANXA6 regulates the MVB‐mediated pathway. However, the underlying mechanism requires further investigation.

Nutrient starvation activates autophagy through stimulating the ULK1 complex‐ULK1, the binding partners ATG13 and FIP200.^75^, ^76^ mTOR negatively regulates the ULK1 complex and autophagy initiation, while inhibition of mTOR activity enhances the ULK1 kinase activity.^75^ Through the quantitative proteomics, there were many ANXA6 candidate interactors that show a connection to mTOR and provide some insight into these observations (Figure 4A). Several membrane‐trafficking RAB and ARF GTPases have also been shown to play key roles in autophagy through the regulation of mTOR signaling.^77^, ^78^ ARF1, localized in the Golgi complex, exerts its function in the expansion of the phagophore through inhibition of mTOR activity.^78^, ^79^ RAB5, localized in early endosomes, regulates PI3K activity and inhibits mTOR‐dependent signaling.^80^, ^81^ GO analysis showed that ANXA6 targets were associated with molecular transport and protein trafficking to induce autophagy. To reveal the role of mTOR signaling in ANXA6‐induced autophagy, we interfered with ANXA6 expression and found that mTOR activity was enhanced, which was mediated by the PI3K‐AKT and ERK signaling pathways (Figure 6). Conversely, the restoration of ANXA6 expression suppressed mTOR signaling with a decrease in phosphorylated S6 and ERK levels. These results demonstrated that ANXA6‐induced autophagy is attributed to the inhibition of mTOR activity.

In summary, we have identified a newly synthesized protein, ANXA6, that is upregulated upon starvation. ANXA6 upregulation is important for inducing autophagy, especially during starvation. The action of ANXA6 has been mainly associated with dysregulation of mTOR, MAPK, and PI3K signaling activities. ANXA6 downregulation is involved in human cervical cancer and may serve as a potential biomarker for the diagnosis, treatment, and prognosis of cervical cancer. ANXA6 displays tumor suppressor effects and ectopic expression of ANXA6 limits the growth of cervical cancer through autophagy induction; thus, their association contributes to cervical carcinogenesis. The possibility of enhancing ANXA6 levels as a cancer treatment is particularly interesting, as ANXA6‐mediated autophagic mechanisms display efficacy in a human cervical cancer xenograft model.

## Supporting information


**FIGURE S1** A, Hela cells with or without *ANXA6* knockdown were treated with rapamycin (100 nM) to induce autophagy. Western blotting was performed to determine the levels of autophagy‐related proteins. B, as in A, cells were treated with rapamycin in the presence or absence of autophagy inhibitor CQ.
**FIGURE S2**. Knockdown of *ANXA6* decreases the formation of autolysosomes. L929‐tfLC3 cells were first transfected with scrambled or *ANXA6* siRNA for 48 h and then treated with rapamycin for 2 h. Confocal microscope was performed to determine the fluorescence intensity (scale bar 10 μm). The number of yellow puncta (GFP^+^RFP^+^) versus red puncta (GFP^−^RFP^+^) was calculated and statistically analyzed. * *P* < .05, ** *P* < .01
**FIGURE S3**. Analysis of ANXA6 targets function and involved pathways. A, GO analysis of the top regulated MF (molecular functions) of ANXA6 targets according to their ranking. B, KEGG analysis of top canonical pathways that the ANXA6 protein targets are significantly over‐represented. C, Representative RAB proteins were listed as target proteins of ANXA6 under normal condition.
**FIGURE S4**. Analysis of localization of ANXA6 in response to starvation. HeLa cells were starved in EBSS for 2 h starvation. The colocalization of ANXA6 with F‐actin was examined using confocal microscope (scale bar 10 µm).
**FIGURE S5**. ANXA6‐induced autophagy involves mTOR signaling pathway. SiHA cells were first transfected with scrambled or *ANXA6* siRNA for 48 h and then starved for 2 h. Cells were harvested and lysed for western blotting. GAPDH served as loading control.
**FIGURE S6**. Knockdown of *ANXA6* accelerates cervical cancer cell growth. A, HeLa cells with or without ANXA6 knockdown were under 24 h starvation. Cells were harvested and labeled with 5 μL Annexin V (Pacific Blue™) and cell fluorescence was measured by flow cytometry. * *P *< .05 B, Cell growth curves in *ANXA6* knockdown cells were drawn with or without rapamycin treatment (200 nM). *** *P *< .001 C, SiHA cells with or without ANXA6 knockdown were treated with rapamycin. Cell proliferation was measured by CCK‐8 and statistically analyzed. * *P *< .05 ** *P* < .01 *** *P* < .001.
**FIGURE S7**. Restoration of ANXA6 expression leads to more cell death under starvation. A, HeLa cells with respective treatments were examined with an inverted microscope and morphological changes were photographed (scale bar 100 µm). B, Quantification of cell apoptosis in *ANXA6* knockdown HeLa cells when ectopic expression of GFP‐ANXA6. Cells were starved in EBSS for 24 h and then labeled with Pacific Blue™ Annexin V for analysis. * *P *< .05 C, SiHA cells with *ANXA6* knockdown were first restored the expression of ANXA6 and then transfected with the *Atg7*‐specific lentiviral shRNA. Cell proliferation was measured by CCK‐8 and statistically analyzed. * *P *< .05 ** *P* < .01 # *P* > .05

Supporting information

Supporting information

## Data Availability

Data available on request from the authors. The data that support the findings of this study are available from the corresponding author upon reasonable request.
