# Supplementary material for: ANXA6 suppresses the tumorigenesis of cervical cancer through autophagy induction
Source: Clin Transl Med. 2020 Oct 19;10(6):e208. doi: 10.1002/ctm2.208 (PMC7571625; doi:10.1002/ctm2.208)

Suppl Figure 1

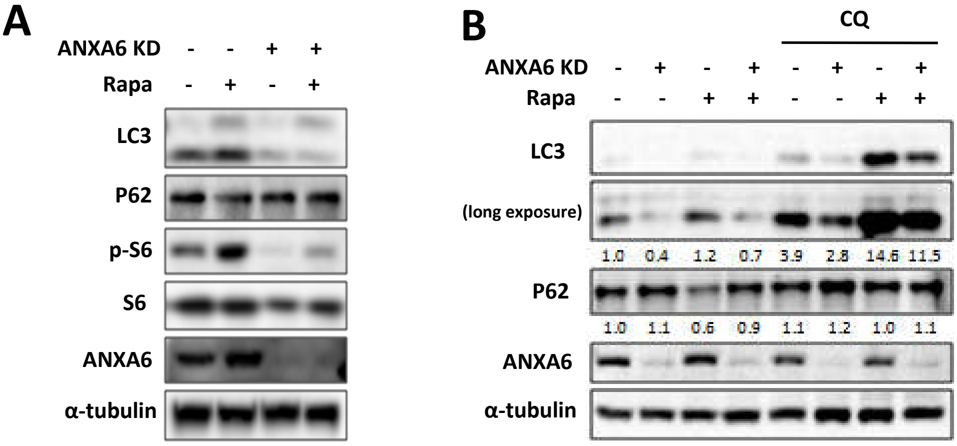

Suppl Figure 2

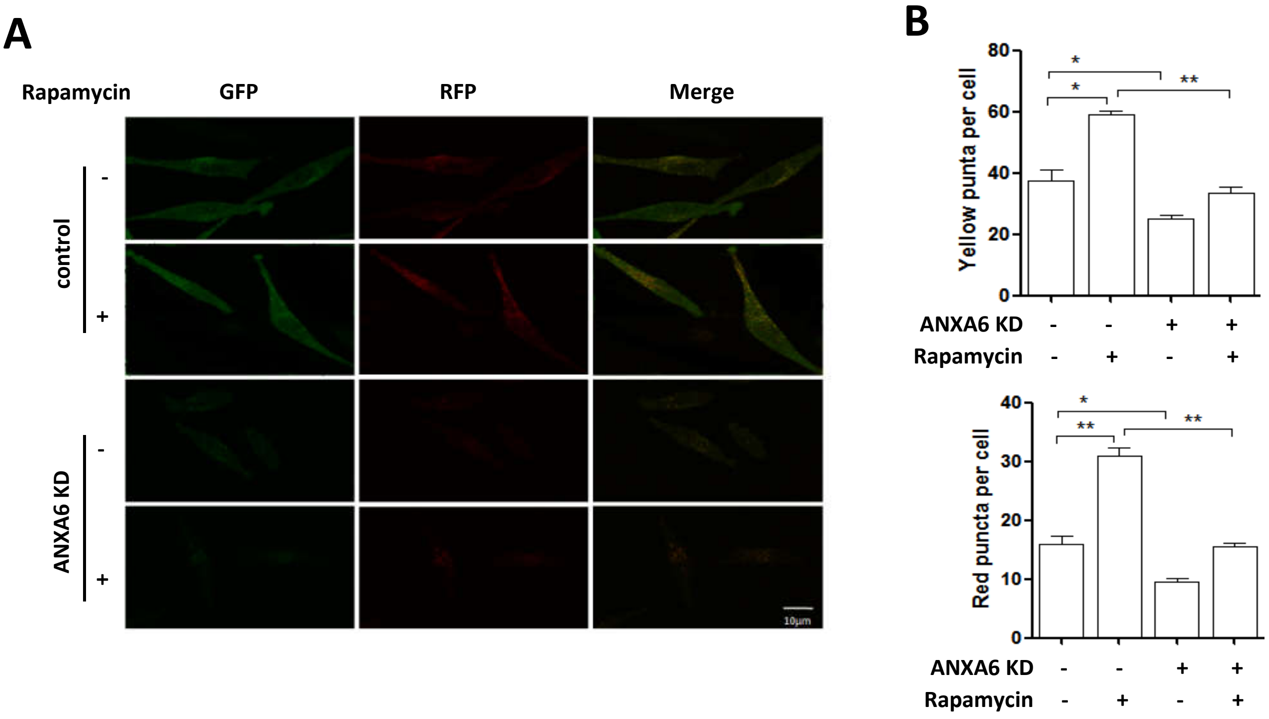

Suppl Figure 3

A

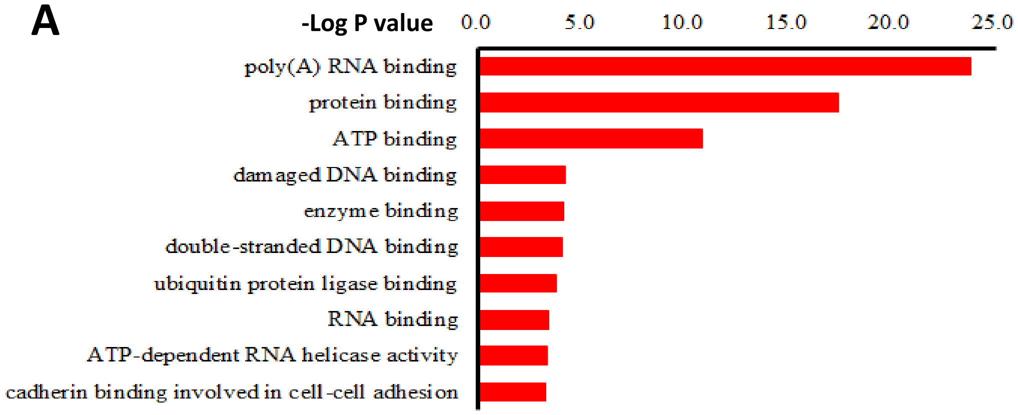

GO analysis of molecular function (MF)

B

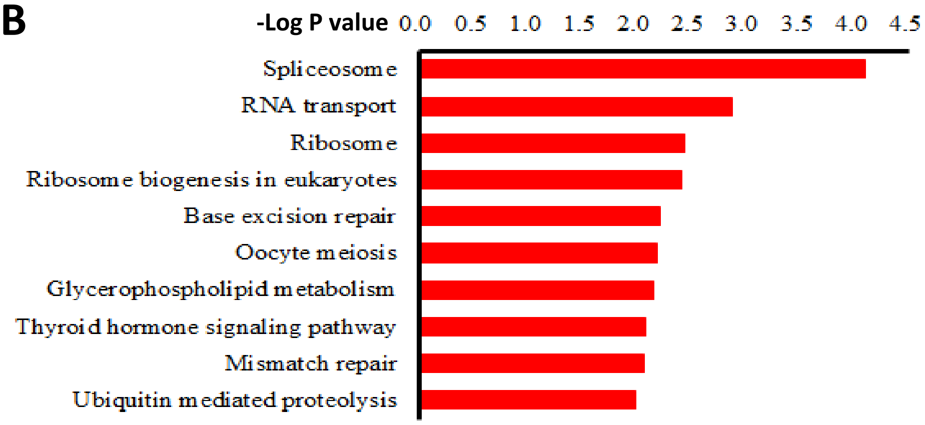

KEGG analysis of pathwasys

C

Protein name	Sum PEP Score	Coverage	Peptide no.
RAB14	175.498	87.90698	16
RAB1B	103.908	73.63184	12
RAB21	86.577	42.66667	10
RAB11	86.577	42.66667	10
RAB8A	55.657	47.82609	10
RAB5	72.378	61.57407	10
RAB34	59.424	51.35135	12
RAB8A	55.657	47.82609	10
RAB10	49.74	48	10
RAB2A	44.732	44.81132	8
RAB35	42.027	48.75622	10
RAB18	32.844	38.83495	7
RAB7	29.475	39.61353	8
RAB6A	18.756	20.19231	4
RAB9A	10.733	20.39801	3

Suppl Figure 4

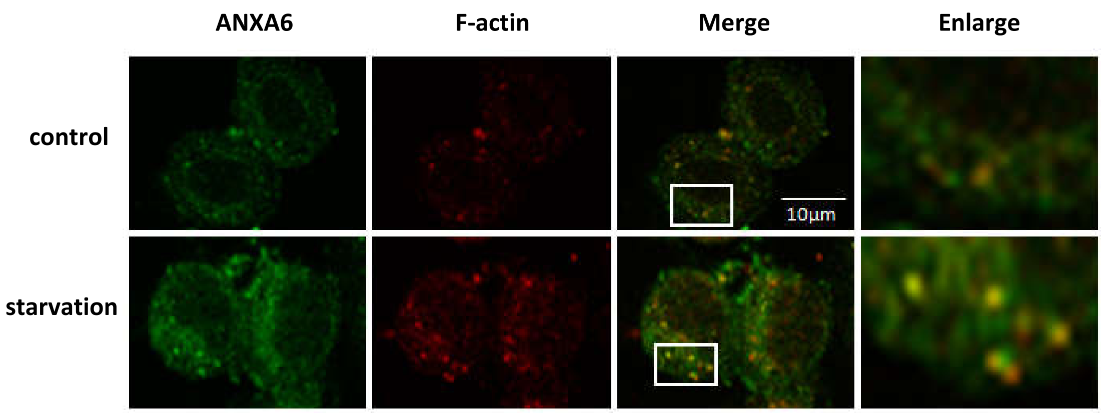

Suppl Figure 5

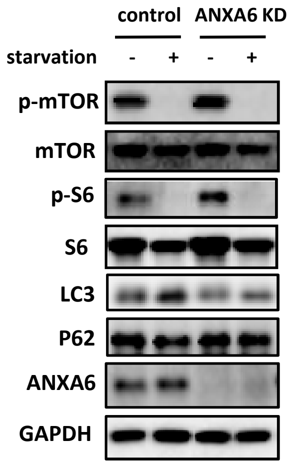

Suppl Figure 6

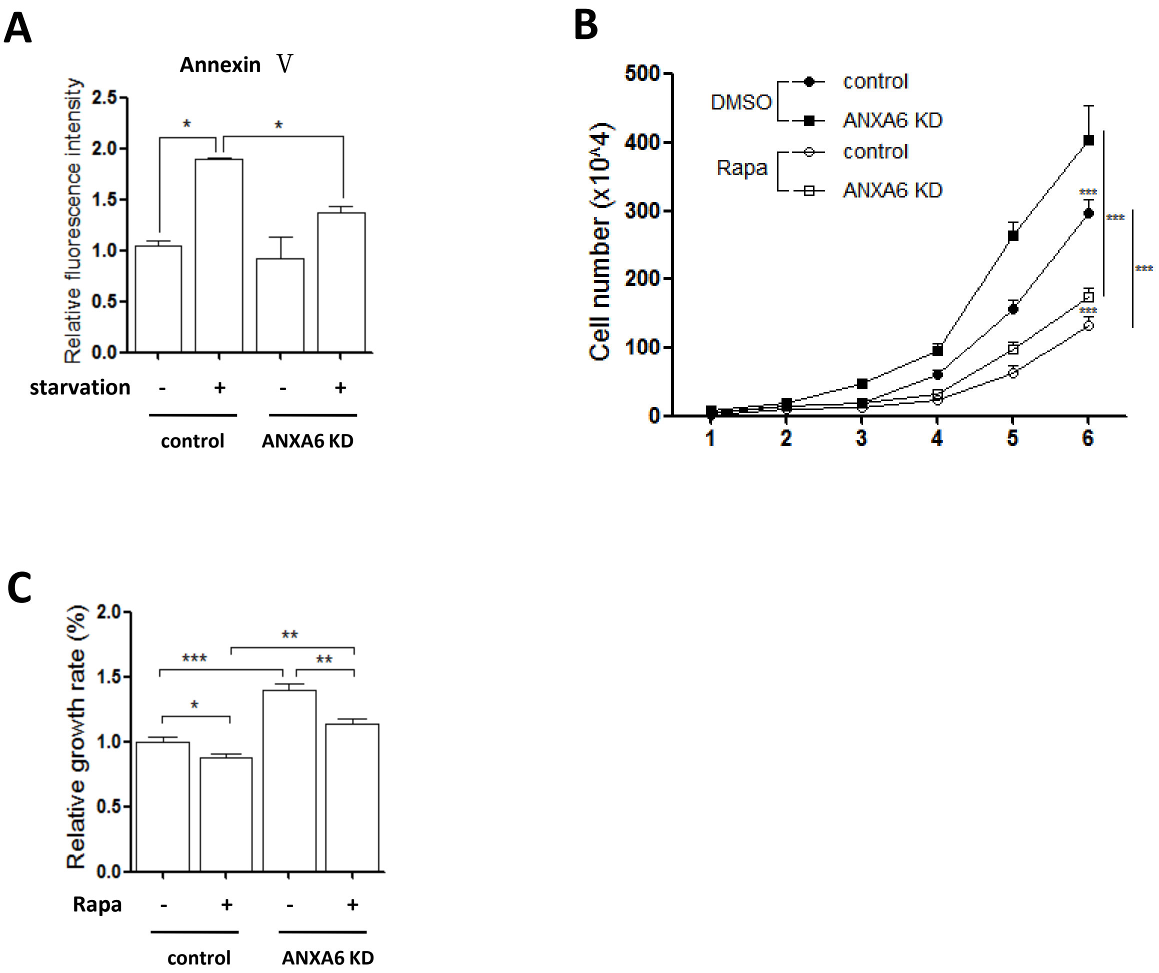

Suppl Figure 7

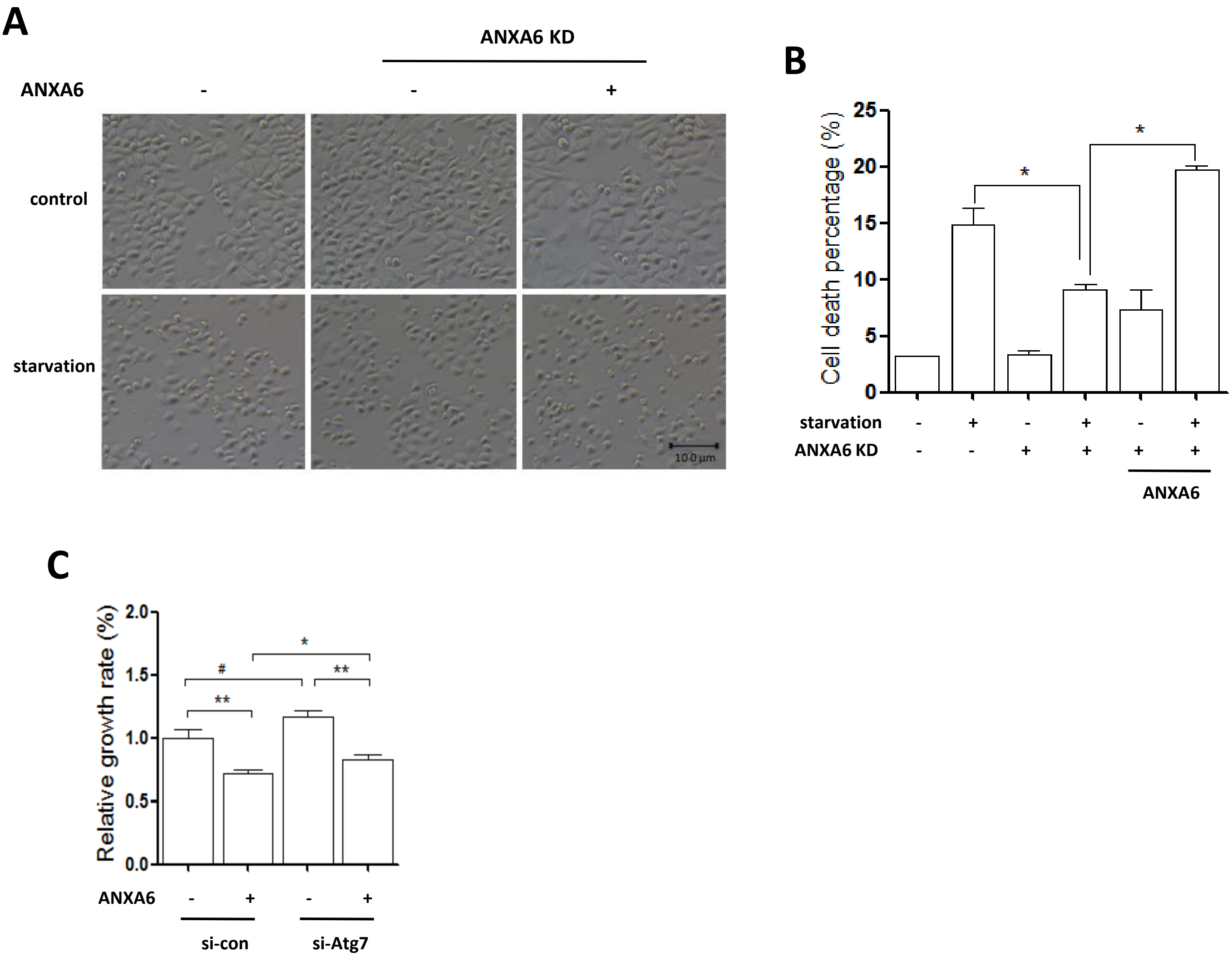

Supplement: Supplementary file 1 — FIGURE S1 A, Hela cells with or without ANXA6 knockdown were treated with rapamycin (100 nM) to induce autophagy. Western blotting was performed to determine the levels of autophagy‐related proteins. B, as in A, cells were treated with rapamycin in the presence or absence of autophagy inhibitor CQ. FIGURE S2. Knockdown of ANXA6 decreases the formation of autolysosomes. L929‐tfLC3 cells were first transfected with scrambled or ANXA6 siRNA for 48 h and then treated with rapamycin for 2 h. Confocal microscope was performed to determine the fluorescence intensity (scale bar 10 μm). The number of yellow puncta (GFP+RFP+) versus red puncta (GFP−RFP+) was calculated and statistically analyzed. * P < .05, ** P < .01 FIGURE S3. Analysis of ANXA6 targets function and involved pathways. A, GO analysis of the top regulated MF (molecular functions) of ANXA6 targets according to their ranking. B, KEGG analysis of top canonical pathways that the ANXA6 protein targets are significantly over‐represented. C, Representative RAB proteins were listed as target proteins of ANXA6 under normal condition. FIGURE S4. Analysis of localization of ANXA6 in response to starvation. HeLa cells were starved in EBSS for 2 h starvation. The colocalization of ANXA6 with F‐actin was examined using confocal microscope (scale bar 10 µm). FIGURE S5. ANXA6‐induced autophagy involves mTOR signaling pathway. SiHA cells were first transfected with scrambled or ANXA6 siRNA for 48 h and then starved for 2 h. Cells were harvested and lysed for western blotting. GAPDH served as loading control. FIGURE S6. Knockdown of ANXA6 accelerates cervical cancer cell growth. A, HeLa cells with or without ANXA6 knockdown were under 24 h starvation. Cells were harvested and labeled with 5 μL Annexin V (Pacific Blue™) and cell fluorescence was measured by flow cytometry. * P < .05 B, Cell growth curves in ANXA6 knockdown cells were drawn with or without rapamycin treatment (200 nM). *** P < .001 C, SiHA cells with or without A [file CTM2-10-e208-s001.pdf]
